# A Microfluidic System for Studying Ageing and Dynamic Single-Cell Responses in Budding Yeast

**DOI:** 10.1371/journal.pone.0100042

**Published:** 2014-06-20

**Authors:** Matthew M. Crane, Ivan B. N. Clark, Elco Bakker, Stewart Smith, Peter S. Swain

**Affiliations:** 1 SynthSys - Center for Synthetic and Systems Biology and School of Biological Sciences, University of Edinburgh, Edinburgh, United Kingdom; 2 Scottish Microelectronics Center, University of Edinburgh, Edinburgh, United Kingdom; University of Toronto, Canada

## Abstract

Recognition of the importance of cell-to-cell variability in cellular decision-making and a growing interest in stochastic modeling of cellular processes has led to an increased demand for high density, reproducible, single-cell measurements in time-varying surroundings. We present ALCATRAS (A Long-term Culturing And TRApping System), a microfluidic device that can quantitatively monitor up to 1000 cells of budding yeast in a well-defined and controlled environment. Daughter cells are removed by fluid flow to avoid crowding allowing experiments to run for over 60 hours, and the extracellular media may be changed repeatedly and in seconds. We illustrate use of the device by measuring ageing through replicative life span curves, following the dynamics of the cell cycle, and examining history-dependent behaviour in the general stress response.

## Introduction

Genetically identical cells may behave in substantially different ways [1]–[3]. The complex, compartmentalized interior of cells and the low copy number of certain cellular constituents ensure cellular processes are inherently stochastic and that variation within an isogenic population is often unavoidable. Such variations can be amplified by genetic circuits and transmitted through time by epigenetic control. Accordingly, stochastic modeling that allows for variation between cells is becoming increasingly important.

To develop quantitative models of cellular behavior, single-cell, time-lapse data is critical[3]–[6]. Conventional approaches such as Fluorescence Activated Cell Sorting (FACS) have been useful in understanding variation in a population at single time points, but are limited by the inability to identify and track the same cells over time. Furthermore, cells grown in batch cultures can experience different environments due to differences in shear stress and variations in cell and resource concentration. Such effects could lead to differential gene expression because of slight differences in nutrients, pH or the degree of competition [7].

An alternative experimental approach is the use of polydimethylsiloxane (PDMS) microfluidic devices. The laminar flow of media in these devices allows the precise control of the environment while cells are observed through time-lapse microscopy. Devices can be made using soft lithography, allowing flow cells to be manufactured reproducibly at relatively low cost [8]. Significant efforts have been made to create microfluidic systems for the culture and observation of single cells, but have focused on designs for mammalian cells[9]–[12].

For studies of *Saccharomyces cerevisiae*, several microfluidic designs have been tried, with varying complexity and experimental application (Table 1). In one of the earliest, cells are adhered within a microfluidic channel to a glass surface coated with the lectin concanavalin-A [13], [14]. The researchers were able to achieve rapid media switching using laminar flow, but were only able to image cells for a few hours. Any regime of fluid flow that removes daughter cells from their mothers also eventually detaches the mothers from the glass. Daughter cells thus accumulate, and after a few divisions the pressure of flow dislodges the entire group of cells. An alternative approach is to constrain cells to grow as monolayers by using chambers of restricted height[15]–[21]. In such devices the daughter cells are retained, and quickly outnumber and surround the original cells. While retaining daughters is useful for some applications, their number can quickly overwhelm a device, limiting the duration of experiments. Furthermore, offspring may affect the local environment of their progenitors, through changing the local flow conditions, consumption of nutrients or release of excreted products. The observation of individual cells over many cell cycles is therefore best achieved in devices that remove progeny.

**Table 1:** Device comparison.

Name/principle	Daughter removal	Media switching	Longest timelapse reported^1^	Single layer deviceand mould	Flow control	Most strains perexperiment ^1^	Most cells per experiment^1^
**Cellasic ** [19]	No	Yes	4h	NA	Air pressure	4	186
**Monolayer ** [20]	No	Yes	24h	Yes	Air pressure	1	20
**Y channel ** [14]	No	Yes	8h	Yes	Media height	1	Not reported
**Microfluidic imaging matrix ** [17]	No	Yes	24h	No	Air pressure/fluidic valves	8	60,000 ^2^
**Microchemostat array ** [15]	No	Yes	24h	No	Air pressure/fluidic valves	1152	1.5×10^8^ ^2^
**Cages ** [22]	Yes	No	18h	Yes	Syringe pump	1	12
**Vertical pressure with biotin/** **avidin ** [23]	Yes	No	Lifespan	No	Syringe pump	1	93
**Pensile columns ** [25]	Yes	No	Lifespan	No	Syringe pump	4	112
**Microfluidic dissection platform ** [24]	Yes	No	Lifespan	No	Syringe pump	1	76
**ALCATRAS 1**	Yes	Yes	36h	Yes	Syringe pumps	1	299
**ALCATRAS 2**	Yes	Yes	67h	Yes	Syringe pumps	1	1003

A comparison of selected commercial and academic devices that have demonstrated single-cell time-lapse cultures for budding yeast. ^1^ Numbers reported refer to the original, cited papers and represent reported data, not theoretical maximum numbers. Some devices, such as the Cellasic, have many citations in which larger numbers of cells may have been recorded and for longer periods. The number of cells refers to those cells originally loaded and excludes daughter cells born in the device except for the Microfluidic imaging matrix and Microchemostat arrays^2^, for which numbers of loaded cells were not reported. Note the trade off between the intervals between imaging frames and the number of cells recorded, and that the density of cells in view has not been reported for most devices. Note also that larger cell numbers can be imaged for shorter times in devices that suffer loss of mother cells over time, such as the ALCATRAS devices. We have assumed that all reported datasets come from a single experiment with a single device.

Three groups have recently demonstrated the removal of *Saccharomyces* daughter cells in microfluidic flow chambers[22]–[25]. One design is based on trapping the mother cells in PDMS cages that have gaps through which budding occurs, leading to removal of daughter cells by the flow of media [22]. Cells are loaded during device assembly, leading to a low occupancy of the traps (<25%). High flow rates and periodic reversal of the flow were required to achieve consistent mother-daughter separation. The other two designs although independently developed are based on the same principle, that of mother cells being compressed from above by PDMS pillars while the smaller daughters are removed by the media flow [24], [25]. These designs allow cells to be observed throughout their lifetime and have provided important insights, particularly in the investigation of cellular ageing.

Here we present a new platform that combines ease of manufacture, environmental switching, high scalability and removal of daughter cells. As an alternative to vertical compression, we designed a device in which cells are held in dense arrays of traps by hydrodynamic pressure. Cells are injected into the assembled device by continuous flow of inoculated media, which allows loading to proceed until more than 90% of traps are filled. New daughters are removed by the flow of the media, and we have imaged over a thousand mother cells. Further, we have imaged dividing cells for up to 67 hours. Media may be switched in a rapid and controlled fashion allowing us to observe individual cell responses to changing external stimuli. We show that cells grown in our device have normal cell cycle times and no signs of stress. In addition, we demonstrate that our device can be used to measure the life span curve of aging cells, the dynamics of the cell cycle, and changes in variation of the stress response during recurring stresses.

## Results

### Microfluidic Traps for Single-cell Imaging

**Figure 1:**
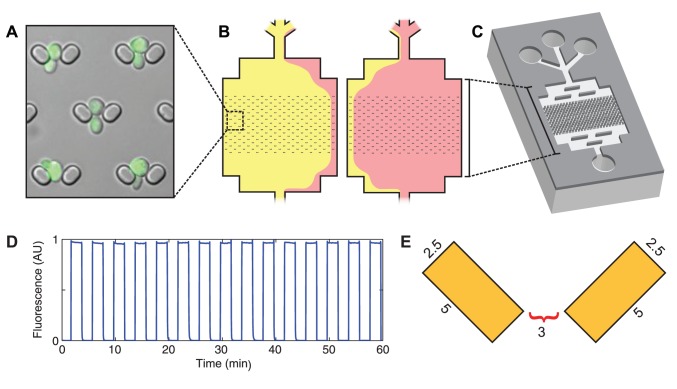
Overview of ALCATRAS. A) A DIC image of the cell traps. The cells are expressing Doa1p-GFP, and the fluorescence image has been overlaid with the DIC image for clarity. Traps consist of two vertical pillars that trap mother cells and allow daughters to flow away. The small size of the traps permits a high density of cells in the field of view (more than 40 mother cells can be imaged at 60x magnification in ALCATRAS 1). B) A schematic depicting fluid flow in the flow cell during media switching. Switching occurs within six seconds by changing the flow rate of syringe pumps. C) Overview of the microfluidic device. Each device contains more than 1500 individual traps. D) The switching rate and reliability of media switching in traps. Media switching was assayed using 0.1% fluorescein in one of the two media. E) Schematic showing trap dimensions in microns.

We designed a novel microfluidic device for studying budding yeast (Fig. 1A–C). The device has three inlet ports upstream of a flow chamber in which the cells are observed. The central port is used for introducing cells; the other two ports are connected to programmable syringe pumps, which drive media flow through the device. Altering the relative flow rates of the pumps allows us to switch the medium in the flow chamber (Fig. 1B,D, movie S1). This chamber contains an array of more than 1,500 individual cell traps, each of which consists of two PDMS pillars that extend from the ceiling of the device to the glass coverslip forming its floor. The media flows through each trap from top to bottom with the pillars oriented such that the gap between the pillars is wide upstream, at the trap entrance, and narrows downstream (Fig. 1E).

**Figure 2:**
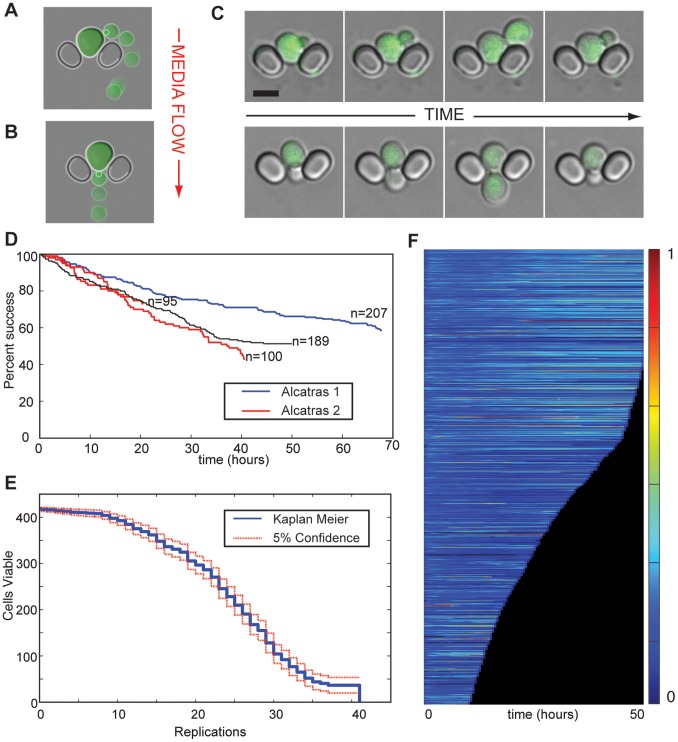
Operation of ALCATRAS. A, B) Schematics showing the removal of a daughter cell by the media flow when the mother buds at the top of the trap (A) or at the bottom (B). In both cases the flow is from top to bottom (red arrow). The newly formed daughter cells follow the streamlines shown in Fig. S1A. C) Microscopy images of removal of daughter cells in the device. The cells are expressing Doa1p-GFP, and the fluorescence image has been overlaid with the DIC image for clarity. Scale bar indicates 5 µm. D) Success rates for four ALCATRAS experiments. The number of cells retained in their original traps over the time course is plotted for two independent experiments using each of ALCATRAS 1 (red – more dense spacing of traps) or ALCATRAS 2 (blue – less dense spacing of traps). Only cells that were present in the traps at the first time point are included. Results were scored manually from a random subset of the fields imaged. Numbers include cells that have visibly died during the experiment. For a more detailed breakdown of cell loss and replacement, see Fig. S3. Flow rates were 2 µl/min from each input syringe pump (4 µl/min total). E) Cell viability plotted as a function of the number of replications (n = 422). Cells were observed for 62 hours, and replicative lifespans were scored manually. The mean lifespan is 22.4. Flow rates were 2 µl/min from each input syringe pump (4 µl/min total). F) Kymograph showing Hsp104-GFP expression over time with imaging every 10 mins. The median fluorescence intensity within the area of each cell (n = 1003) at each time point is shown by the colour map. Only cells that are present during the first hour of the experiment and that remain in their original traps for at least 10 hours are shown.

Refinements of this basic design have produced a simple device in which daughters are efficiently removed from the mothers with a low rate of failure (Fig. 2). Once a cell is caught, the hydrodynamic resistance of the trap increases: diverting the fluid flow (Fig. S1A) and creating a pressure differential across the cell from the top to the bottom of the trap (Fig. S1B). This low energy pocket, combined with viscous forces from fluid flow, prevents a cell from being pulled out of the trap. Diversion of the streamlines around trapped cells also creates a positive feedback loop during cell loading: cells bypass occupied traps in preference for empty ones, rapidly filling the array. This same process aids removal of daughter cells from the device during operation.

Daughter cells are removed by the flow in two ways depending on the polarity of the trapped mother cell (Fig. 2A–C). If the bud is oriented towards the top of the trap, the diverted flow moves the bud to the side and later removes the resulting daughter cell (Fig. 2A, C, movie S3). If the bud forms downstream of the mother, it is extruded between the pillars and separates rapidly from the mother on completion of cytokinesis (Fig. 2B,C, movie S2). Mother cells in this position usually continue to bud through the gap because haploid cells form successive buds in close proximity [26]. The dimensions of the cell trap were optimized over numerous design cycles. Each design was evaluated for loading and retention of mother cells (Fig. S2).

Using the best performing design we created two alternative devices (ALCATRAS 1 and ALCATRAS 2) that differ in the spacing between adjacent traps and characterized their ability to retain mother cells in time-lapse experiments (Fig. 2D, Fig. S3, device details in figures S4 and S5). Of the two devices, ALCATRAS 1 has narrower spacing between traps, which allows more cells to be included in each field of view on the microscope (Fig. S2). An alternative device in which cells are held by vertical pressure allows approximately 30% of the original cells to be retained in view at 20 hours of observation [24]. After 20 hours of culture in ALCATRAS 1, approximately 70% of cells remain trapped within the device (Fig. 2D, S3). This number is an improvement of over 200% compared with any published device for which such data are available.

As cells age and approach senescence, they often become enlarged and have larger daughters. In our devices these large cells can become trapped by the limited height of the flow chamber, leading to device crowding, which may divert media flow and eventually pushes mother cells out of some traps. Consequently, the length of time for which over 1000 cells may be imaged is long (approx. 36 hours in ALCATRAS 1) but limited. We find that such device crowding is greatly delayed in ALCATRAS 2, the device with more widely spaced traps (Fig. S2E & movie S4). There is also an increase in cell retention (Fig. S3). This device therefore performs better in long time-lapse experiments, and, after 60 hours, clogging has only become a problem in 25% of the fields of view (Fig. S2E). Defining a clog as a group of 50 or more touching cells, the mean running time before the appearance of one clog is 24.6 hours (S.E 13.3, n = 5), the earliest appearance of a clog was after 12.3 hours and one experiment (out of 6 considered) was run for 58 hours without any clogs.

To demonstrate the advantages of the device for studying ageing, we monitored the reduction in cell viability as a function of replicative age, a commonly used measure of lifespan [27]. Using a wild-type (S288C) strain, we grew cells in 2% glucose in XY media, imaged cells for over 62 hours in the device, and then manually scored the viability as a function of replicative age (Fig. 2E). In a single device, we measured the replicative lifespans of over 400 cells. The life span curve shows strong agreement with those obtained using either other microfluidic systems or conventional micro-dissection with a mean replicative lifespan of 24.7 divisions using the Kaplan-Meier estimator [23], [24], [27], [28].

Further, we have recorded the level of the heat-shock protein Hsp104p-GFP, a marker for protein damage, as a function of age [29]. In a single experiment, we acquired images from over 1,000 cells at 10-minute intervals and over a 50-hour period (Fig. 2F, movies S4 and S5). Only cells that were present during the first hour of the experiment and that remained in a trap for at least 10 hours were analysed (Fig 2F). This result represents nearly an order of magnitude increase in cell numbers over reports using other devices in which daughter cells are also removed (Table 1).

### Cell Cycle Dynamics Appear Normal in the Microfluidic Device

**Figure 3:**
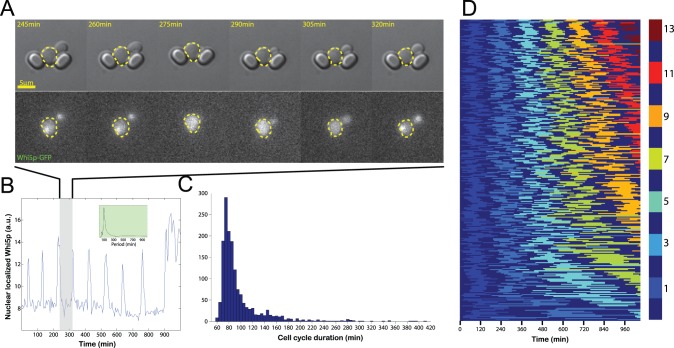
Cell cycle dynamics in ALCATRAS. A) Frames from a time-lapse movie showing a cell expressing Whi5p-GFP across one cell cycle. Fluorescence is localized in the nucleus during late M and early G1 phases. B) Plot of the nuclear localization of Whi5p over time for the cell shown in A. Frames in A are from the shaded period. Over many hours the cell undergoes a large number of cell cycles, resulting in a strongly periodic signal. The inset shows the power spectrum derived from applying Welch’s windowing algorithm and the Fourier transform to this data with a single peak at the frequency of the cell cycle. C) Histogram showing the distribution of cell cycle times of mother cells undergoing their first three divisions in ALCATRAS. D) Kymograph illustrating the change in replicative age of individual cells during the experiment. To aid visualization of the cell cycle, each alternate division is marked by a different colour. The total number of divisions undergone in the device when odd is shown by the colour map; even numbers of division are depicted in dark blue. Cells have been ordered by their number of divisions, and only cells that remain alive and in their original traps throughout are illustrated (299 cells). Flow rates were 2 µl/min from each input syringe pump (4 µl/min total).

For a cell culture platform to be useful, it is essential that cellular behaviour is not significantly disturbed under the conditions imposed. We have addressed this concern by investigating the growth of cells in ALCATRAS (Fig. 3). To characterize the dynamics of cell division, we have imaged cells expressing a Whi5p-GFP fusion, which is localized in the nucleus during late M and early G1 phases and is distributed throughout the cell during the remainder of the division cycle [30] (Fig. 3A). Quantification of nuclear-localized GFP in trapped mother cells therefore provides a periodic signal (Fig. 3B), from which we can extract statistics on the divisions of each cell. Over the first three divisions after loading the device, the mode of the cell cycle times is 75 minutes, similar to results from mother cells physically constrained by a low PDMS ceiling (71 minutes [24]) and faster than times measured for mother cells seeded onto agarose slabs (87 minutes [31]). Any environmental stresses generated by cell loading or the flow conditions do not therefore delay the cell cycle.

With the 100s of cells that we tracked in this experiment, we can observe the de-synchronization of cell cycles from one cell to another and a wide distribution of cell cycle times in the population, as previously noted [31] (Fig. 3C,D). Further, our data are consistent with ageing affecting the cell cycle: after 5 divisions, the distribution of cell division times is statistically different from the distribution of division times for the first division (pairwise Kolmogorov Smirnov test, p = 0.012, comparing 1^st^ division with 6^th^ division which have means of 97.6min and 106.5min, respectively).

The normal growth that we observe in the device indicates that cells either do not experience significant stress or respond to a stressful environment in ways that allow division at typical rates. To distinguish between these possibilities, we have observed localization of GFP fused to the general stress marker Msn2p, which migrates to the nucleus in response to many different stresses [32], [33]. We observe only sporadic nuclear localization of Msn2-GFP, similar to observations of unconstrained cells (Fig. S6 and data not shown), indicating that the general stress response pathway is not activated as a result of culturing cells in our device.

### Learning in the General Stress Response

Thriving in the constantly changing environments that cells typically inhabit requires an ability to identify and respond to stresses. A single transcription factor (Msn2p) is involved in the activation of hundreds of different stress response genes[32]–[35]. Msn2p has unusual dynamics, with alternative modes of nuclear shuttling that lead to activation of different subsets of genes [36]–[38]. Responding appropriately to stress involves *prima facie* careful regulation. A cell that fails to sense a significant and increasing stress could fail to respond and thus be removed from the gene pool. Alternatively, stress responses cause the mobilization of large numbers of proteins. If a cell responds to a stress that fails to materialize fully, it would be disadvantaged relative to cells that did not, paying the cost of the response yet receiving no benefit.

**Figure 4:**
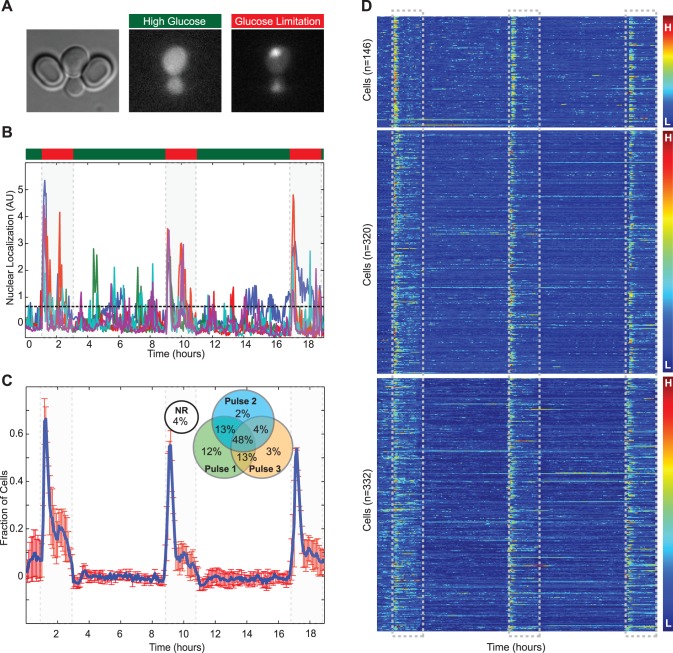
Stress response and learning. A) An image of a cell in ALCATRAS expressing Msn2p-GFP. Under conditions of high glucose, the majority of signal is localized to the cytoplasm, but almost immediately upon glucose limitation becomes nuclear localized. B) Nuclear localization of five single cells under repeated glucose limitation. Media was repeatedly switched from high glucose (2% - green) for 6 hours to low glucose (0.1% - red) for 2 hours. The dashed line shows the threshold for classification of Msn2p-GFP as being nuclear localized. C) Fraction of cells with nuclear localized Msn2p-GFP (n = 3 experiments, error bars are SEM.) (Inset) A Venn diagram showing the fraction of cells that respond for each of the three pulses over all experiments. The difference in number of cells responding to each of the three pulses was statistically significant (p<0.01). D) A kymograph showing the single cell traces and results for all cells (n = 146, 320, 332). Flow rates were 4.5 µl/min (dominant pump) and 0.5 µl/min. The rates were reversed for media switching which was confirmed by inclusion of cy5 dye in one of the media reservoirs.

Even if cells could sense the external environment perfectly, they may respond differently depending on their own history and intracellular state. Imaging cells over tens of divisions and providing repeated stimuli is an opportunity to observe how individual cells adapt and modulate their responses to stress. As proof of principle for the capabilities of ALCATRAS for such experiments, we observed the response of cells to repeated limitations of glucose. When cells that are exposed to high glucose (2%) suddenly experience a low glucose environment (0.1%), Msn2p-GFP becomes nuclear localized [33], [36], [37] (Fig. 4A). The cell thus recognizes this nutrient limitation and activates a stress response using a single period of sustained nuclear localization followed by more stochastic localizations. The cells were grown to log phase in 2% glucose, and then introduced to the device. We switched the media to low glucose for 2 hours and then to high glucose for 6 hours. This sequence was repeated three times, and the responses of individual cells varied substantially (Fig. 4B, D). Trends did emerge when the cells were viewed as a collective (Fig. 4C). Compared to the first glucose limitation, the fraction of cells with nuclear Msn2p-GFP is reduced during the second and third limitations (Fig. 4C). We classified cells as having nuclear localized Msn2p if the nuclear localization was greater than the population mean of all cells over the whole time course plus one standard deviation. After each limitation, cells were scored as having responded if Msn2p is nuclear localized within two standard deviations of the mean population response time.

Most cells respond to the first stimulus (87%), but fewer respond to the second (67%) and third (68%) (Fig 4C inset). The reduction in the fraction of responsive cells at each limitation is statistically significant (p<0.01). This reduction could be a result of learning: if a temporary stress fails to be sustained, cells are less likely to respond to the same stress in the future. Alternatively, the reduction could result from ageing, and a reduced ability to sense and respond appropriately to extracellular changes.

To distinguish between these two possible mechanisms we performed separate experiments with young and old cells with identical life histories. Cells were placed in the device and allowed to grow in high glucose for either 2 hours or 18 hours and the media was then switched once. The fraction of cells responding to the stress is not statistically significant (87% of young cells and 89% of old cells, p>0.05), suggesting that the decrease in the number of cells responding to repeated limitations of glucose is a result of life history and not senescence. The role of such memory in cellular responses is only beginning to be explored, but this exploration will be enabled with microfluidic devices such as ALCATRAS.

## Discussion

The microfluidic device we present is the first that combines the removal of daughters with the ability to change the external environment of cells, allowing us to follow individual cell responses to different stimuli over tens of replications. Although there are now several microfluidic systems for imaging of *Saccharomyces* cells (Table 1), this study demonstrates a novel method for removing daughter cells that can be incorporated into future designs.

Our devices have several advantages over other strategies for daughter cell removal. The design is quick, simple and inexpensive to manufacture, requiring a single patterned mask to create a single layer mold. Devices are attached to a glass coverslip using standard techniques. Designs based on vertical constriction of cells require molds created by multiple spin-coating, mask alignment and lithography steps[23]–[25], and a similar but alternative design based on PDMS cages requires a sapphire coverslip held to the device in a custom-made holder [22]. ALCATRAS traps dense arrays of cells so that large numbers are visible in each microscopic field. Devices that hold cells by vertical pressure contain pensile columns or micropads, each of which covers hundreds of square microns yet typically hold only one or two mother cells [24], [25]. The numbers of cells in each field of view is therefore severely restricted. Since stage speed and the time for acquiring each image or stack limit the number of fields that can be followed, the increase in cell density in ALCATRAS results in a direct increase in the total number of cells that may be recorded. For this reason, we have been able to raise the scale of data collection by an order of magnitude (Table 1).

To gather large datasets, a device must also be loaded efficiently. Our devices can be fully loaded using flow conditions similar or identical to those used during data collection. Alternative devices are either loaded at low efficiency [22] or require special conditions such as high pressure or device centrifugation, which have the potential to affect cell behaviour[21], [23]–[25].

Our device opens up new possibilities for biological research. The ability to retain mother cells while measuring responses to environmental change will allow studies of the physiology of ageing. Precise experimental control of the cellular environment will allow interrogation of the mechanisms by which cells process signals, and so provide insight into cellular decision-making and determination of cell fate. In systems biology, the ability to obtain time-series of thousands of cells will improve model fitting and selection. Such large datasets, coupled with environmental control, may also allow studies of rare events, such as the emergence of drug resistance. The focus of cell biology research is shifting from the study of populations to the study of individuals. Our microfluidic device and others like it have the potential to make single-cell studies widely available and statistically robust.

## Materials and Methods

### Device Fabrication

The device was fabricated using conventional soft-lithography techniques [39], [40]. Device designs were created using AutoCad software (Autodesk) and printed (Compugraphics) to a chrome-on-glass mask. A ∼6 µm tall mold was fabricated at the Scottish Microelectronics Centre using SU8 3005 photoresist. Mold heights were measured using a profilometer following fabrication. To prevent elastomer sticking, a thin layer of Trichloro(1*H*,1*H*,2*H*,2*H*-ng)silane (Sigma-Aldrich) was evaporated onto the wafer before molding. Devices were made using a 10∶1 mixture of Sylgard 184 (Dow Corning) and curing agent and cured at 70**°**C. Following fabrication, holes were punched to allow fluid access and devices were bonded to coverslips following oxygen plasma treatment.

CAD files are available on request and will be made available at swainlab.bio.ed.ac.uk.

### Yeast Strains and Cell Preparation

All strains used were from the yeast GFP clone collection, which was created in the S288C genetic background [41]. For experiments, a single colony was placed into a liquid culture of synthetic complete (SC) medium and grown overnight. Cells were cultured in 2% glucose in SC media, and then diluted and allowed to grow to log phase before being introduced to the device. The replicative life span experiment used XY media (2% peptone, 1% yeast extract, 0.01% adenine, and 0.02% tryptophan) and only cells that remained in the device for over 45 hours were scored. These cells were scored based on number of replications, and the Kaplan Meier estimator was used to determine the mean replicative lifespan.

### Data Acquisition and Analysis

All experiments were performed on a Nikon Eclipse Ti inverted microscope controlled using custom Matlab scripts (Mathworks) written for Micromanager [42]. An incubation chamber (Okolabs) was used to maintain the microscope and microfluidic device at a constant temperature of 30°C. Experiments used either a 60X 1.2NA water immersion objective or a 100X 1.4NA oil objective (Nikon). Images were acquired using an Evolve EMCCD camera (Photometrics) with a 512 x 512 sensor. Media was delivered using two syringe pumps (New Era Pump Systems, model NE100), located in an incubator set at 30°C and set at a total flow rate of 4 µl/min throughout the extended imaging period. The Nikon Perfect Focus System (PFS) was used during all experiments to maintain accurate focus over many hours. The ALCATRAS 1 device was used for the cell-cycle experiments and the first Msn2p experiment where cells experienced repeated glucose limitation. All other experiments used ALCATRAS 2.

In switching experiments, cells close to the edges of the device are excluded from consideration because diffusion of signaling molecules may occur in the vicinity of the media interface. A fluorescent dye (cy5, gift of C. Portal) was added to one of the media sources to monitor the exact timing of media switching in experiments using cells, while 0.1% fluorescein (BDH) was used during device characterization (Fig 1D and movie s1). Switching speed is limited by capacitance in the system, the result of using non-rigid materials, including the plastic syringes, polytetrafluoroethylene PTFE tubing, and the PDMS device itself. Switching speeds were improved significantly by including a capacitance-correction step in the pump programs. On initiation of switching, the previously-dominant pump is set to withdraw at a high speed for a limited time, while the new dominant pump perfuses at the same high speed for the same time. This correction rapidly eliminates capacitance upstream of the device, allowing a dramatic increase in switching speeds without affecting the flow rate experienced by the trapped cells. Using this protocol the media was changed in the cell trapping area within 6s (Fig 1D).

Image segmentation, cell tracking and data extraction was performed using customized code in Matlab (Mathworks). Traps were tracked through time using an approach based on normalized cross-correlation. Individual cells were identified with a trained classifier using a support vector machine. Following identification of a cell, the outline was detected using a circular Hough transform.

For both cell cycle and glucose limitation experiments the nuclear GFP localization was determined by the protocol of Cai *et al*. [38], which is unaffected by photobleaching and changes in numbers of fluorophores. For the cell cycle experiments, we applied a threshold to the rise in nuclear localization between successive time points to identify nuclear entry of Whi5p-GFP. For the glucose limitation experiments, we calculated the threshold for determining whether a cell had nuclear localized Msn2p-GFP using the mean and standard deviation of all cells for each experiment. To calculate the fraction of cells that responded to each glucose limitation, we determined the mean response time to the first limitation, and the standard deviation of the response time. The fraction that responded to each limitation (Fig 4C inset) was determined by the fraction that responded within two standard deviations of the mean. The statistical significance of the change in the number of cells responding after each shock of low glucose was assessed by a chi-squared test with the null hypothesis that the response of each cell for each of the three shocks was a sample drawn from the same binomial distribution, the probability of success of which was estimated as the mean response probability of the pooled data for all three shocks. This hypothesis was not supported (p<0.01).

## Supporting Information

Figure S1 **A) Simulations of a finite element model of unoccupied and occupied cell traps showing streamlines.** As traps become filled, the hydrodynamic resistance of the traps increases and the streamlines change. The velocity surface is shown by the colormap and units are in µm/s. B) Model showing the pressure drop across an occupied and an unoccupied trap. Trapped cells alter the pressure drop and create a low energy pocket that helps keep cells trapped. Modelling involved solution of the 2-dimensional incompressible Naviers-Stokes equation at steady-state using Comsol Multiphysics 4.3 (Stockholm, Sweden) and the laminar flow physics library. The finite element model was based on the Autocad design file used to create the mold and a physics controlled mesh size with 35,000 elements. The mesh density was such that increasing the number of elements did not affect convergence of the solution. No slip conditions were used for all boundaries except the inlet (top) and outlet (bottom), and a 10 Pa pressure drop was used for the simulation.
(EPS)

Figure S2 **Design iterations.** Quantification of the characteristics of the traps and their affects on the performance of ALCATRAS. A) Schematic of yeast trap showing the trap length and gap between pillars. B) Density of traps at 60X magnification on our microscope. C) Increasing the trap length leads to both an increase in the initial trapping rate, and the fraction of cells that are retained after 10 hours. D) Modulating the space between the pillars results in different percentages of cells being trapped and held. E) Reducing the density of traps within a single field of view significantly increases the time that the device can be used for imaging without clogging. A device is classified as clogged when >25% of the fields of view have 50 or more cells touching each other. ALCATRAS1 has a gap of 3 µm, length of 4.5 µm, 44 traps per field of view. ALCATRAS 2 has a gap of 3 µm, length of 5 µm and 28 traps per field of view.
(EPS)

Figure S3 **Cell fates in ALCATRAS 1 and 2.** Quantification of the retention of cells, cell death in traps, cell loss from traps and replacement of cells in traps from two independent experiments in each of ALCATRAS 1 and 2. Data were scored manually from DIC movies from a sample of imaging fields. Fields showing significant clogging were excluded. The two ALCATRAS 1 experiments are shown by the x and+symbols and the ALCATRAS 2 data by triangles. The proportion of live cells still present is shown in blue; the cumulative number of cells that have visibly died in traps in black; the cumulative number of cells that have been lost from the traps in green; and the cumulative number of cells that have been replaced (in successive time points) in red. Fig. 2d shows the number of cells that have died plus the number of cells that were retained and alive using the same data.
(EPS)

Figure S4 **ALCATRAS 1 design.** Schematic showing the design of ALCATRAS 1 with high trap density that allows for many cells to be imaged, but also increases the propensity to clog.
(EPS)

Figure S5 **ALCATRAS 2 design.** Schematic showing the design of ALCATRAS 2 with slightly increased spacing between traps that results in a lower density of cells to be imaged, but also reduced propensity to clog.
(EPS)

Figure S6 **Cells behave normally in the device.** A) Average Msn2p response of cells experiencing high glucose and no stress (n = 84). The cells consistently show a low average response. B) Kymograph showing the single cell traces.
(EPS)

Movie S1 **Media switching in ALCATRAS.** One media inlet has been filled with 0.1% fluorescein distilled water and the other with distilled water. Media was switched within 6 seconds.
(AVI)

Movie S2 **Daughter cell budding (bottom).** Movie showing how a daughter cell is removed when budding towards the bottom of the trap (downstream). The majority of cells bud in this fashion.
(MOV)

Movie S3 **Daughter cell budding (top).** Movie showing how a daughter that buds towards the direction of flow is removed.
(MOV)

Movie S4 **Imaging >1000 cells in ALCATRAS 2.** Single movie created from 55 microscope fields stitched together from a 67-hour experiment using ALCATRAS 2. Each field was imaged at 10 minute intervals using a 60x objective. DIC images are shown. Extensive device clogging occurs from 50 hours onwards. Media flow is from left to right. Cells are Hsp104p-GFP. movie S5 shows the GFP channel.
(AVI)

Movie S5 **Fluorescence imaging in ALCATRAS 2.** Movie created from 55 microscope fields showing Hsp104p-GFP expression of cells in ALCATRAS 2 (the same experiment as movie S4). Data from this movie is shown in Fig. 2E.
(AVI)
